# The Effect of Physical Activity and the Use of Active Video Games: Exergames in Children and Adolescents: A Systematic Review

**DOI:** 10.3390/ijerph17124243

**Published:** 2020-06-14

**Authors:** Irwin Andrés Ramírez-Granizo, José Luis Ubago-Jiménez, Gabriel González-Valero, Pilar Puertas-Molero, Silvia San Román-Mata

**Affiliations:** 1 Department of Didactics of Musical, University of Granada, 18071 Granada, Spain; irwinrg@ugr.es (I.A.R.-G.); jlubago@ugr.es (J.L.U.-J.); pilarpuertasmolero@gmail.com (P.P.-M.); 2 Nursing Department, University of Granada, 18071 Granada, Spain; silviasanroman@ugr.es

**Keywords:** exergames, physical activity, children, systematic review

## Abstract

The aim of this study is to develop a systematic review on the relationship between the use of active video games “exergames” and the practice of physical activity. The Web of Science (WOS) repository was used as the main search engine, using as criteria the selection of longitudinal and experimental studies published in the last five years. A total of eight research papers were obtained, in which intervention programs based on the use of exergames were applied to improve different parameters, such as adherence to Physical Activity practice or improvement on a psychological level. As the main findings, it was possible to observe the need to include these types of devices in the classroom since they can work transversally across much content, and the resources are so accessible that they allow improvements at academic level. Likewise, they favor motivation to physical exercise since with adequate volume and intensity parameters, they are related to healthier lifestyles, and the areas of motor skills and logical thinking benefited the most.

## 1. Introduction

In current society, lifestyles have changed significantly among children, becoming increasingly negative and harmful to their health [1]. The time spent on physical activity has been reduced by other habits such as the well-known sedentary digital leisure, which are related to high rates of sedentarism and child obesity [2,3,4]. This, together with the consumption of ultraprocessed foods and foods rich in fat, salt and sugar, causes an increase in the infant’s weight levels, thus producing severe pathologies at an early age [5]. 

Modern environments and technological advances have radically altered the way people live. The need for physical activity is no longer paramount but a mere pleasure or hobby. Physical activity as a means of survival has disappeared due to the constant evolution of patterns, society and means, thus causing sedentary behavior that has accommodated us positively in certain aspects but negatively in many others. Information and communication technologies (ICTs) have become a real addiction for young people, perpetuating sedentary behaviors and contributing to excessive time in front of them [6]. This means that physical inactivity predominates over this type of hobby, leading to an increase in the hours of use of this type of device and its replacement by recreational or sporting activities. Authors such as Jakes [7], mention that time spent in front of a computer, tablet, video game or electronic device seems to be directly related to overweight and obesity rates in children. In order to address this problem, it is essential to increase PA levels, understood as any body movement that involves energy expenditure [8]. International organizations suggest for young people at least 60 min a day with moderate intensity and a high aerobic component [9]. In this sense, González-Valero [10] points out the benefits of physical exercise at a multifactorial level. On the one hand, at the physical level, it has been revealed that higher levels of PA are associated with better body composition, greater bone density or high sensitivity to insulin. In addition, cognitive benefits suggest that continuous sport practice prevents stress and anxiety, while it improves self-esteem or attention span along with executive functions [11].

Along these lines, new technologies can help us by serving as a support and complement, but never as a substitute for physical activity [12]. Exergames or active video games are presented as the perfect complement to encourage physical activity focused on the interests of children because of the games’ dynamics, their presentation and also because they generate a different motivation and stimulation in each person that is sufficient to improve PA levels [13]. This type of device has become the technological focus of PA, which supplies the needs of users at the moment of exercise, stimulating them through play and physical activity competence [14]. The suppression of peripherals such as the keyboard, controller and mouse, allows the user to move in a real environment and simulate it within the game or virtual reality, where players without the need to be in a passive position (sitting) use movements, body gestures, as well as voice to develop in the different video games. While the use and interpretation of gestures allows for great applicability in different scenarios (leisure, rehabilitation, training, error correction), emerging technologies used by large companies such as Sony or Microsoft make this a competition to satisfy user needs [4]. Positive affective aspects, such as intrinsic motivation, centers of interest, and the power to enjoy a particular activity pleasantly, are shown to be powerful predictors of PA over time [15]. This perspective makes it clear that a review of the existing literature on this subject is necessary, especially in longitudinal and experimental studies, since opposing and contradictory results are observed.

In this sense, the aim of the study was to carry out a systematic review of scientific literature addressing the effect of PA practice and the use of exergames through longitudinal and experimental studies.

## 2. Materials and Methods 

This review followed the guidelines of the PRISMA statement for systematic reviews in order to ensure an adequate structure and development of the document [16] and complied with the items proposed by Sotos-Prieto [17] in which it mentions the points to be taken into account for the realization of a systematic review.

### 2.1. Search Strategy and Procedure

The database used to carry out the proposed systematic review was the Web of Science (WOS). The SCOPUS search engine was also used in order to contrast the information obtained in the main database. The review was conducted during the month of February 2020, analyzing studies that addressed the physical condition and use of exergames in school-aged children. The period of this search was from 2016 to 2020, using as keywords “*Physicalactivity*”, “*Exergames*”, and *“Children*” and using “*and*” as the Boolean operator. In the refinement of the search, all publications written in English from the “Main Collection of Web of Science” that were in the research domain “*Education Educational Research*” and “*Sport Sciences*”were considered. Following these guidelines, 62 research papers were obtained.

**Figure 1:**
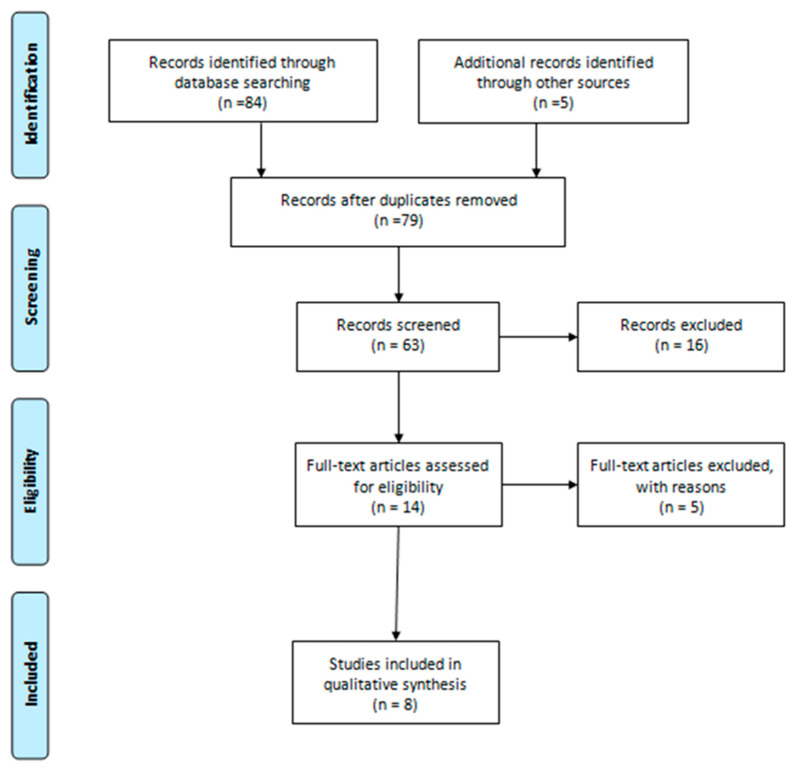
Flowchart of the selection of the base body of study.

The inclusion criteria to define the set of research works that are part of the study sample were (1) scientific studies that present PA variables and the use of exergames; (2) research that resorts to a longitudinal design; (3) research that shows statistical results that allow the analysis of the study variables. As can be seen in Figure 1, for the identification phase, inclusion criterion number 1 was taken into account (scientific studies presenting PA variables and the use of exergames). Later, in the screening phase, it was evaluated that it met criteria 1 and 3 (scientific studies that present PA variables and the use of exergames, and research that shows statistical results that allow the analysis of the study variables). For the eligibility phase, criteria 2 and 3 were taken into account (research using a longitudinal design and research showing statistical results that allow the analysis of study variables).

### 2.2. Population and Sample of Scientific Literature

The population of scientific papers set for this study was 79 documents extracted from the WOS data repository. The sample that composed the base body of the following systematic review corresponds to 8 scientific publications, considered after applying the inclusion and coding criteria (Figure 1).

## 3. Results

This section shows the descriptive results of the selected studies (*n*=8) that address the improvement of physical activity practice through exergames.

### 3.1. Evaluation of Scientific Production

Based on the above procedure and search strategy, a total of 15 scientific research articles on the influence of physical activity and exergames were registered in WOS during the period 2016–2020, considering “education educational research” as the main research area. In relation to the product obtained globally from scientific literature on this subject in WOS, this study represented 46.6% (*n*= 7) of the global computation. Reviewing the total production, a growing trend can be observed since 2016, reaching a peak in 2017 with 23 publications. A decrease is observed in 2019 with 14 scientific papers, although it should be noted that this cycle was open when the review was carried out in February/April 2020. In relation to the body of the study, it was shown that production increased between 2016 and 2017, and decreased in 2018 with four publications, so it could be established that there was a decrease in publications that analyzed the influence of PA practice through the use of exergames in school-aged children in the last two years.

### 3.2. Results of Studies Selected for Systematic Review

Table 1 shows the results obtained after the systematic review applying the search criteria described above and analyzing the association between PA practice and the use of active video games in school-aged children, pre-adolescents, and adolescents. If the sample of each study is agglutinated, a total of 1004 subjects between the ages of 6 and 16 years old were obtained. The majority of the studies participated in longitudinal studies of diverse typology with some type of control in order to verify the causal relationships between different modes of physical activity practice through the use of electronic devices (active video games) and diverse factors linked to the practice of sports, such as heart rate, reaction speed, and motivation towards physical activity. For the organization of the information in the systematic review, the following coding was followed: (1) authors and years of publication; (2) the methodological design; (3) sample and its breakdown into an experimental or control group (GC or GE); (4) minimum and maximum age; (5) basic description of the intervention carried out; (6) duration of the intervention; (7) variables considered in the study; (8) instruments used for the assessment of the variables; and (9) conclusions and findings.

## 4. Discussion

The main conclusions obtained from the systematic review carried out on the basis of the longitudinal and experimental studies evaluated are shown. The body of the study consists of seven scientific papers with randomized designs that address the issue of exergames and their influence on PA practice in pre-adolescents. For this purpose, different platforms, games, and active dynamics are used, where active electronic devices are used to promote healthy lifestyles and multi-component improvements, among others.

### 4.1. Exergames

It was determined that the use of video games is widespread, as indicated by Chacón-Cuberos [18], where 9 out of 10 students used some type of electronic platform for either digital or even active leisure. This indicates the great center of interest around these devices, which are very attractive to schoolchildren due to their ease of use and the stimulation they provide when played. Additionally, it was seen that a small part of this sample confessed to substitute activities and a third of the total sample confessed to grumpiness if they could not play, showing disorders in daily life due to the addiction to them. With respect to its use as a complement to PA, it was possible to verify how those participants who tried these devices were positioned in favor of it within the area of physical education, due not only to the recreational component but also to the use of it as a tool for performing physical exercise with effects similar to daily or traditional sports [19].

### 4.2. Physical Condition and General Health

Most of the experimental studies analyzed considered, along with the effect of exergames, the level of physical activity and its influence on health. In this regard, it was observed that, regardless of the active video games used, they can never replace moderate or vigorous physical activity, but can help to improve levels of sedentariness or encourage a more playful way of learning certain content of the different subjects [18,19], obtaining improvements in participation, healthy parameters, such as heart rate, or improvements even in terms of body health [19,20].

### 4.3. Cognitive and Academic Performance

Cognitive performance also benefits from sports or physical activity. When using electronic devices, the tasks employed and played on them require cognitive demands involving gross motor skills that have a higher effect [25]. This also allows for an improvement in brain irrationality, concentration, attention, and reaction speed, although no improvement in executive functions is seen [22,25].

### 4.4. Limitations of the Study

Finally, it is essential to highlight the main limitations of this systematic review. To begin with, the search range can be highlighted, which can be considered both a weakness and strength. The studies used were limited to the last five years in order to provide an updated view of the state of the art. However, such a narrow time interval may have missed some studies of national or international relevance. Another limitation may lie in the exclusive selection of “longitudinal” and “experimental” studies. As with the previous limitation, this selection criterion helps to generate conclusions that are determined by causality.

### 4.5. Transfer Effects

None of the studies selected in this review investigated the possible transference effect of cognitive training with video games for activities of daily living. These studies only raise the possibility that the effects of training can be extended to everyday tasks using the same neural networks. However, to our knowledge, there are no studies investigating the transference effect of skills developed through video game training on healthy adults in real life [26,27,28,29,30,31,32,33].

## 5. Conclusions

It can be seen how cognitive performance and speed of reaction are benefited by the practice of PA using these devices. When using this type of stimuli, there is greater cognitive demand and therefore it implies a higher gross motor effect.

Most studies prove that, independently of the degree of PA and the long-term effect it can produce, improvements are produced in terms of motivation, health status and participation of students, especially through sports games where the motor demand is higher. There are no major changes in body composition but there are changes in heart rate.

Thus, it is shown how the use of active video games is an innovative element to encourage the practice of physical activity at an early age as long as they are complemented by frequent or intense sports activities. It also highlights the importance of promoting healthy lifestyles in order to combat sedentariness and states of obesity, diabetes, cholesterol, or cardiovascular problems.

Finally, very disparate results were observed in most of the studies, generally due to the existence of the wide variety of contexts, instruments used, duration and methodologies. For this reason, it is necessary to unify criteria to evaluate the variables analyzed.

## Figures and Tables

**Table 1:** Papers that address exergames and physical activity in children.

Research	Design	Sample (E–C)	Age (min–max.)	Intervention	Duration	Variables	Instrument	Conclusions
Research 1. [18]	Comparative and cross-sectional descriptive study	520 (520–ND)	8–12	Ad Hoc Fulfillment	15 min	Exergames in physical education Video Console Frequency Fatigue Physical Exercise	Test Ad Hoc (8 items)	The data show that primary school pupils show a favorable attitude to the use of exergames in physical education.
Research 2. [19]	Longitudinal cross study	124 (124–ND)	6–12	3 sessions (20 min each)	1 hour	Experience exergame Heart rate Gender	Microsoft Kinect video game	Exergames can raise a student’s CF from 11 to 18 years to a moderate level of intensity when assigned as part of the physical education curriculum.
Research 3. [20]	Controlled and randomized intervention	61 (32–29)	10–ND	Teaching activity with the use of mobiles (15 min)	4 weeks	Effectiveness exergames Motivation towards learning	Validated scale Semi-structured interview	The learning effect was stronger with the video game than the traditional study method, but the longitudinal learning effects of the game have yet to be verified.
Research 4. [21]	Controlled and randomized intervention	20 (20–ND)	(7–16)	3 sessions of 60 min each of playing Just Dance 1/2	2 months	Physical Activity Range and forms of movement	Direct observation	The use of exergames as a pedagogical tool can improve the knowledge of students in the movement, which could lead to a fruitful pedagogical collaboration.
Research 5. [22]	Measurements pre-post test repeated in one group	30 (30–ND)	(7–12)	30 min game x 3 stations	6 months	Processing and execution time		The use of exergames can complement physical education programs, or substitute for sedentary activities while at home.
Research 6. [23]	Intervention controlled and randomized	62	(12–16)	Investigate whether exploring a 3D video game environment stimulates the hippocampus	1 month (1.5 h each)	Examine the cognitive and neural underpinnings of two different types of game learning in order to evaluate their common and separate correlates, with the hope of informing future intervention research	JHU atlas	Correlation between the learning of video games and white matter was evidenced. There was cognitive improvement of working memory and perception.
Research 7. [24]	Intervention controlled and randomized	126 (126–ND)	(10–15)	Play with Wii platform (Wii Fit and Nintendo games)	6 weeks (30 min game)	Physical condition Self-EfficacyApoyo social percibido	PAQ-C BMI Technology use frequency questionnaire	The use of exergames is a type of exercise and sport friendly intervention that can improve the physical condition and attitude of adolescents towards physical activity.
Research 8. [25]	Controlled and randomized intervention	123 (62–61)	(11-ND)	Use of motor actions in math class	8 weeks (3 sessions each subject) 24 total sessions	Reaction speedCognitive performance	“Math Performance Test” Juego Kinesético The D2 test	It was emphasized that digital games can be very powerful learning tools that students will always find interesting Math-based exergames had a positive effect on students’ mental computation speed.
